# Follow-up after embolization of ruptured intracranial aneurysms: A prospective comparison of two-dimensional digital subtraction angiography, three-dimensional digital subtraction angiography, and time-of-flight magnetic resonance angiography

**DOI:** 10.1007/s00234-012-1030-z

**Published:** 2012-04-10

**Authors:** Zbigniew Serafin, Piotr Strześniewski, Władysław Lasek, Wojciech Beuth

**Affiliations:** 1 Department of Radiology and Diagnostic Imaging, Nicolaus Copernicus University, Collegium Medicum, ul. M. Skłodowskiej-Curie 9, 85-094 Bydgoszcz, Poland; 2 Department of Neurosurgery and Neurotraumatology, Nicolaus Copernicus University, Collegium Medicum, ul. M. Skłodowskiej-Curie 9, 85-094 Bydgoszcz, Poland

**Keywords:** Intracranial aneurysm, Therapeutic embolization, Cerebral angiography, Magnetic resonance angiography, Follow-up studies

## Abstract

**Introduction:**

To prospectively compare of the diagnostic value of digital subtraction angiography (DSA) and time-of-flight magnetic resonance angiography (TOF-MRA) in the follow-up of intracranial aneurysms after endovascular treatment.

**Methods:**

Seventy-two consecutive patients were examined 3 months after the embolization. The index tests included: two-dimensional DSA (2D-DSA), three-dimensional DSA (3D-DSA), and TOF-MRA. The reference test was a retrospective consensus between 2D-DSA images, 3D-DSA images, and source rotational DSA images. The evaluation included: detection of the residual flow, quantification of the flow, and validity of the decision regarding retreatment. Intraobserver agreement and interobserver agreement were determined.

**Results:**

The sensitivity and specificity of residual flow detection ranged from 84.6 % (2D-DSA and TOF-MRA) to 92.3 % (3D-DSA) and from 91.3 % (TOF-MRA) to 97.8 % (3D-DSA), respectively. The accuracy of occlusion degree evaluation ranged from 0.78 (2D-DSA) to 0.92 (3D-DSA, Cohen’s kappa). The 2D-DSA method presented lower performance in the decision on retreatment than 3D-DSA (*P* < 0.05, ROC analysis). The intraobserver agreement was very good for all techniques (*κ* = 0.80–0.97). The interobserver agreement was moderate for TOF-MRA and very good for 2D-DSA and 3D-DSA (*κ* = 0.72–0.94).

**Conclusion:**

Considering the invasiveness of DSA and the minor difference in the diagnostic performance between 3D-DSA and TOF-MRA, the latter method should be the first-line modality for follow-up after aneurysm embolization.

## Introduction

In many centers, endovascular embolization has become a method of choice for the treatment of intracranial aneurysms [1, 2]. However, coiled aneurysms present a significant rate of recanalization, which occurs in approximately 20 % of patients [3, 4]. Due to the possibility of recanalization and the availability of relatively safe endovascular retreatment [5], follow-up of coiled aneurysms is recommended [6, 7].

Despite the invasiveness, need for hospitalization and relatively high cost, intra-arterial digital subtraction angiography (DSA) is still the standard follow-up method after aneurysm embolization [7–9]. Recently, several reports have indicated that DSA can be replaced by magnetic resonance angiography (MRA), which is less invasive and presents very good accuracy in detecting residual flow in the aneurysm [6, 10–12]. However, several authors have indicated that in some cases of aneurysm recanalization, MRA may be more sensitive than the commonly used two-dimensional DSA (2D-DSA) [11, 13–15]. Some other authors have suggested that three-dimensional DSA (3D-DSA) may be more sensitive in detecting residual flow than 2D-DSA [16, 17]. This raised a question of contemporary standard for follow-up imaging of embolized aneurysms.

The purpose of the study was a prospective comparison of the diagnostic value of 2D-DSA, 3D-DSA and time-of-flight MRA (TOF-MRA) at follow-up regarding the determination of aneurysm occlusion and the decision-making process regarding possible retreatment.

## Materials and methods

### Population

The study was approved by our university’s review board and was performed in accordance with the Declaration of Helsinki. All participants provided written informed consent. The sample size calculation was based on a meta-analysis by Kwee and Kwee [18], who estimated the pooled sensitivity and specificity for TOF-MRA in detecting aneurysmal flow at 83.3 % (95 % confidence interval [CI], 70.3–91.3 %) and 90.6 % (95 % CI, 80.4–95.8 %), respectively. Using the method of Flahault et al. [19] and assuming a possible 5 % rate of non-evaluable cases, the sample size was estimated at 74 aneurysms.

We included patients who were treated for subarachnoid hemorrhage due to aneurysm rupture, that were scheduled for the first follow-up imaging at 3 months after the procedure. Patients were excluded for the following reasons: (a) age under 18 years, (b) contraindications to MR imaging, including severe claustrophobia, ferromagnetic foreign bodies or electronic implants, (c) the presence of neurosurgical clips, (d) estimated glomerular filtration rate (eGFR) <60 ml/min/1.73 m^2^.

Embolizations were performed using platinum coils (GDC Detachable Coils, Boston Scientific, Natick, MA, USA; Axium and Nexus, ev3 Corporate, Plymouth, USA; MicroPlex, MicroVention, Inc., Tustin, USA), hydrogel coils (HydroCoil and HydroSoft, MicroVention, Inc., Tustin, USA), and intracranial stents (Neuroform3; Boston Scientific). Intracranial stents were used for coiling assistance in patients with wide-neck aneurysms to achieve a better packing density and to prevent coil prolapse into the parent artery. After all of the procedures, the baseline status of the aneurysm was documented by 2D-DSA and 3D-DSA.MR imaging was not routinely performed during the perioperative period.

### Follow-up DSA technique

Intra-arterial DSA was performed with a monoplane angiographic unit (Axiom Artis dTA, Siemens Medical Systems, Erlangen, Germany) by means of transfemoral catheterization. Contrast material (iopromide, Ultravist 300 mg-I/ml; Bayer Schering Pharma AG, Berlin, Germany) was administrated with a power injector through a 5 F catheter. The 2D-DSA acquisition consisted of three projections: posteroanterior (LAO/RAO 0 °, CRAN 26 °), oblique (LAO/RAO 26 °, CRAN 26 °), and lateral (LAO/RAO 90 °, CRAN 0 °). Contrast agent was administered at 12 ml (6 ml/s) to common carotid arteries (CCA) and at 10 ml (5 ml/s) to vertebral arteries (VA).

The 3D-DSA imaging included arteries with embolized aneurysms only. The acquisitions consisted of two rotational scans, covering 200 °, resulting in 122 2D-source images in cine mode. Contrast agent was administered at 15 ml (5 ml/s) to CCA and at 8 ml (3 ml/s) to VA. Images were analyzed on a dedicated workstation (Syngo XVP VA72B, Siemens AG, Berlin, Germany) using InSpace 3Dsoftware. The following reconstruction parameters were used: voxel size 0.57 mm, number of slices 220, slice matrix 512 × 512, kernel type EE, reconstruction mode Dual Volume.

### Follow-up MRA technique

MR angiography was performed with a 1.5-T Signa Hdx unit, using an eight-channel HD Brain Coil (GE Medical Systems, Waukesha, USA) within 24 h after DSA. A 3D TOF ASSET Multislab technique was used in axial plane to cover the whole intracranial space. The following parameters were used: TE 2.7 ms, TR 30 ms, flip angle 20 °, bandwidth 31.25 kHz, section thickness 1.2 mm, matrix 320 × 224, effective voxel size 0.7 × 0.8 × 0.6 mm.

Angiograms were evaluated with Advantage Workstation 4.4 and Volume Share 8.4.3 software (GE Medical Systems). Analysis included non-reconstructed images, as well as MIP and VR reconstructions.

### Image analysis

Examined methods were evaluated as concerns the detection of residual flow in the aneurysm, classification of the flow, i.e., classification of the degree of aneurysm occlusion with a method described by Roy et al. [20], and possibility of retreatment.

Index tests included 2D-DSA, 3D-DSA, and TOF-MRA. Images were independently assessed by two interventional neuroradiologists (Z.S, P.S.) with a 10-year experience in the field. The observers evaluated blinded data, and were unaware of the other imaging results of the patient. Discordant results were solved by means of joint reassessment.

The reference result was established retrospectively when results of index tests were set in all study participants. The reference test was DSA, which constituted a simultaneous analysis of 2D-DSA images, 3D-DSA VR images, and source rotational DSA images. Examinations were evaluated by two observers and all discrepancies between methods were solved by consensus.

### Statistical analysis

Test characteristics of 2D-DSA, 3D-DSA, and TOF-MRA versus the reference method were calculated with corresponding 95 % CIs. We tested the ability of the index tests to properly detect residual flow in the aneurysm and to properly define indications for retreatment. The test characteristics included sensitivity, specificity, positive predictive value (PPV), negative predictive value (NPV), positive likelihood ratio (LR+), negative likelihood ratio (LR−), and diagnostic accuracy. We also calculated the areas under the receiver operating characteristic curves (AUCs) with their 95 % CIs. Significance of the AUC values and significance of differences between them was tested with *z*-test. The ability of the index tests to properly classify the degree of aneurysm occlusion (class 1–3) was estimated using concordance correlation coefficient (CCC) with its 95 % CI and weighted Cohen’s kappa (*κ*). Intraobserver agreement and interobserver agreement were measured with Cohen’s *κ* with its 95 % CI. A *P* value of <0.05 was considered significant. Statistical analyses were performed using MedCalc 11.6.0 (MedCalc Software, Mariakerke, Belgium) and Statistica 9 (StatSoft Inc., Tulsa, OK, USA).

## Results

**Fig. 1:**
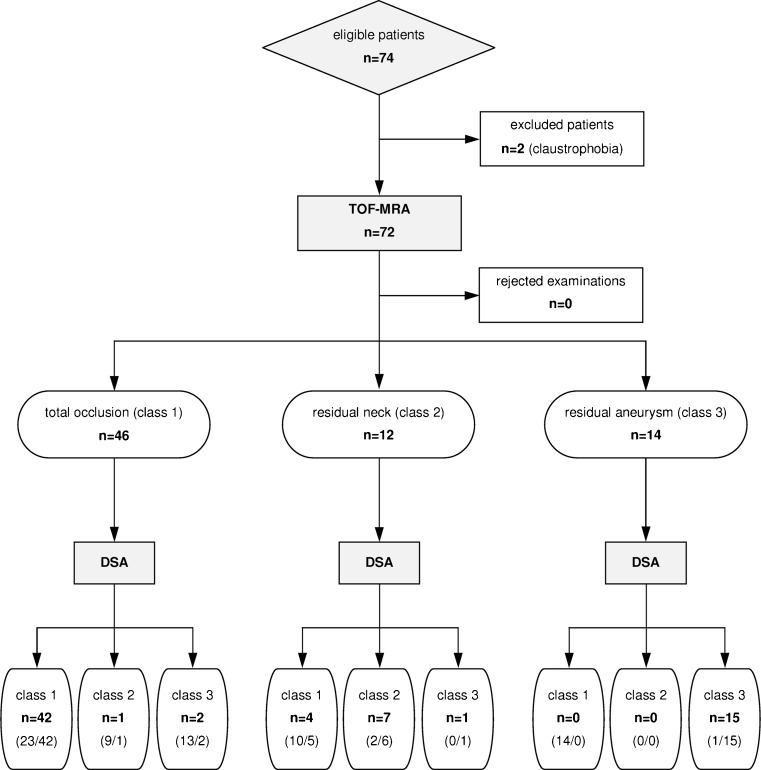
Flow diagram presenting the process of patient recruitment and results of imaging. Fields report numbers of aneurysm assessments with TOF-MRA and DSA (reference test). Fields concerning DSA include also results of 2D-DSA/3D-DSA imaging (in *parentheses*)

Between November 2009 and March 2011, a total number of 74 patients with 74 aneurysms were prospectively included in the study (Fig. 1). Due to a severe claustrophobia two patients were excluded from the MRA group. Therefore, 72 patients (mean age 51.5 ± 12.4 years) including 24 men and 48 women were finally analyzed (Table 1). During follow-up examinations no adverse reactions to contrast media occurred and no adverse events related to DSA were noted. The mean radiation dose of 2D-DSA in three projections was 97 ± 14 mGy, and the mean radiation dose of 3D acquisition was 102 ± 12 mGy.

**Table 1:** Baseline characteristics of included patients with percentages in parentheses

Characteristic		Value
Aneurysm location	ICA	40 (55.6 %)
	ACoA/ACA	17 (23.6 %)
	MCA	10 (13.9 %)
	BA/VA	5 (6.9 %)
Largest aneurysm diameter	small (≤5 mm)	26 (36.1 %)
	medium (5.1–15 mm)	42 (58.3 %)
	large (15.1–25 mm)	5 (6.9 %)
	giant (>25 mm)	0 (0.0 %)
Sack/neck ratio	≤1.5	15 (20.8 %)
	1.6–2.5	36 (48.6 %)
	>2.5	21 (29.2 %)
Number of coils placed	≤ 3	15 (20.8 %)
	4–6	25 (34.7 %)
	> 6	32 (44.4 %)
Coiling method	Platinum coils	48 (66.6 %)
	Mixed coils^a^	24 (33.3 %)
	Stent-assisted	8 (11.1 %)
Result of embolization^b^	Class 1	64 (89.9 %)
	Class 2	8 (11.1 %)
	Class 3	0 (0.0 %)
Vasospasm after embolization		9 (12.5 %)

*ICA* internal carotid artery, *ACoA* anterior communicating artery, *ACA* anterior cerebral artery, *MCA* middle cerebral artery, *BA* basilar artery, *VA* vertebral artery

^a^Platinum and hydrogel coils

^b^According to Roy at al. [20]

Follow-up DSA images were interpretable in all the cases. The reference test presented residual flow in 26 aneurysms (36.1 %): class 2 in eight cases (11.1 %), and class 3 in 18 cases (25.0 %). Technical indications for retreatment were found in 12 patients. When comparing the follow-up results to the immediate post-treatment imaging, we found that: 45 aneurysms remained unchanged and 15 aneurysms recanalized or presented progression of the residual flow. In two cases, the initially observed residual necks occluded spontaneously.

**Fig. 2:**
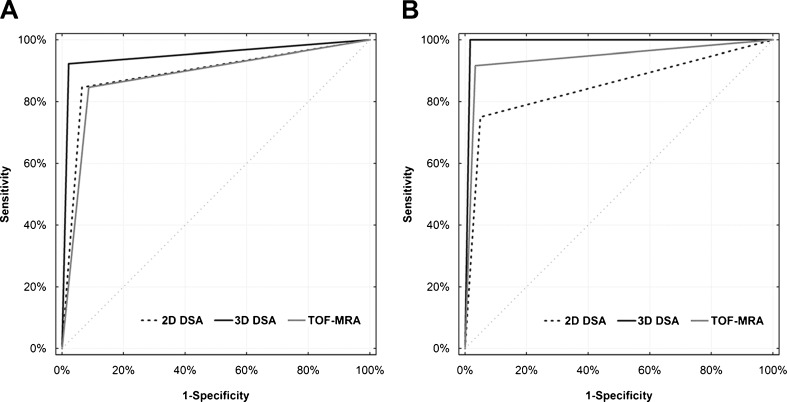
Receiver operating characteristic curves for **a** the detection of residual flow in the aneurysm and **b** decision making on possible retreatment

Characteristics of index tests are presented in Tables 2 and 3, and in Fig. 2. The 2D-DSA correctly diagnosed complete occlusion in 43 patients and residual flow in 22 patients, while three diagnoses were false-positive and four were false-negative. Determination of the class of occlusion was incorrect in 12 patients. The decision on retreatment based on 2D-DSA was true-positive in cases, false-positive in three cases, false-negative in three cases, and true-negative in 57 patients. Detection of the residual flow in 3D-DSA was true-positive in 24 cases, false-positive in one case, false-negative in two cases, and true-negative in 45 patients. Estimation of the occlusion class was incorrect in three patients. The 3D-DSA correctly defined indications for retreatment in all but one case, which was false-positive.

**Table 2:** Comparison of test characteristics of 2D-DSA, 3D-DSA, and TOF-MRA in the detection of residual flow in the aneurysm (values with 95 % CIs in parentheses)

Characteristic	2D-DSA	3D-DSA	TOF-MRA
Sensitivity (%)	84.6 (65.1–95.5)	92.3 (74.8–98.8)	84.6 (65.1–95.5)
Specificity (%)	93.5 (82.1–98.6)	97.8 (88.4–99.6)	91.3 (79.2–97.5)
PPV (%)	88.0 (68.8–97.3)	96.0 (79.6–99.3)	84.6 (65.1–95.5)
NPV (%)	91.5 (79.6–97.6)	95.7 (85.4–99.4)	91.3 (79.2–97.5)
LR+	13.0 (10.8–15.5)	42.5 (37.70–47.8)	9.7 (8.1–11.7)
LR−	0.16 (0.04–0.70)	0.08 (0.01–0.80)	0.17 (0.05–0.60)
Accuracy (%)	90.3 (83.3–97.1)	95.8 (91.2–100.0)	88.9 (81.6–96.2)
AUC	0.89 (0.80–0.95)	0.95 (0.87–0.99)	0.88 (0.78–0.94)

*PPV* positive predictive value, *NPV* negative predictive value, *LR +* positive likelihood ratio, *LR −* negative likelihood ratio, *AUC* area under the receiver operating characteristic curve

**Table 3:** Comparison of test characteristics of 2D-DSA, 3D-DSA, and TOF-MRA in the decision-making process regarding possible retreatment (values with 95 % CIs in parentheses)

Characteristic	2D-DSA	3D-DSA	TOF-MRA
Sensitivity (%)	75.0 (42.8–94.2)	100.0 (73.4–100.0)	91.7 (61.5–98.6)
Specificity (%)	95.0 (86.1–98.9)	98.3 (91.0–99.7)	96.7 (88.4–99.5)
PPV (%)	75.0 (42.8–94.2)	92.3 (63.9–98.7)	84.6 (54.5–97.6)
NPV (%)	95.0 (86.1–98.9)	100.0 (93.9–100.0)	98.3 (90.9–99.7)
LR+	15.0 (10.8–20.9)	60.0 (58.1–62.0)	27.5 (23.0–32.8)
LR−	0.26 (0.06–1.20)	0.00 (0.00–0.01)	0.09 (0.018–0.90)
Accuracy (%)	91.7 (85.3–98.1)	98.6 (95.9–100.0)	95.8 (91.2–100.0)
AUC*	0.85 (0.75–0.92)	0.99 (0.93–1.00)	0.94 (0.86–0.98)

*PPV* positive predictive value, *NPV* negative predictive value, *LR +* positive likelihood ratio, *LR −* negative likelihood ratio, *AUC* area under the receiver operating characteristic curve

^*^Significant difference between 2D-DSA and 3D-DSA (*P* < 0.05)

**Fig. 3:**
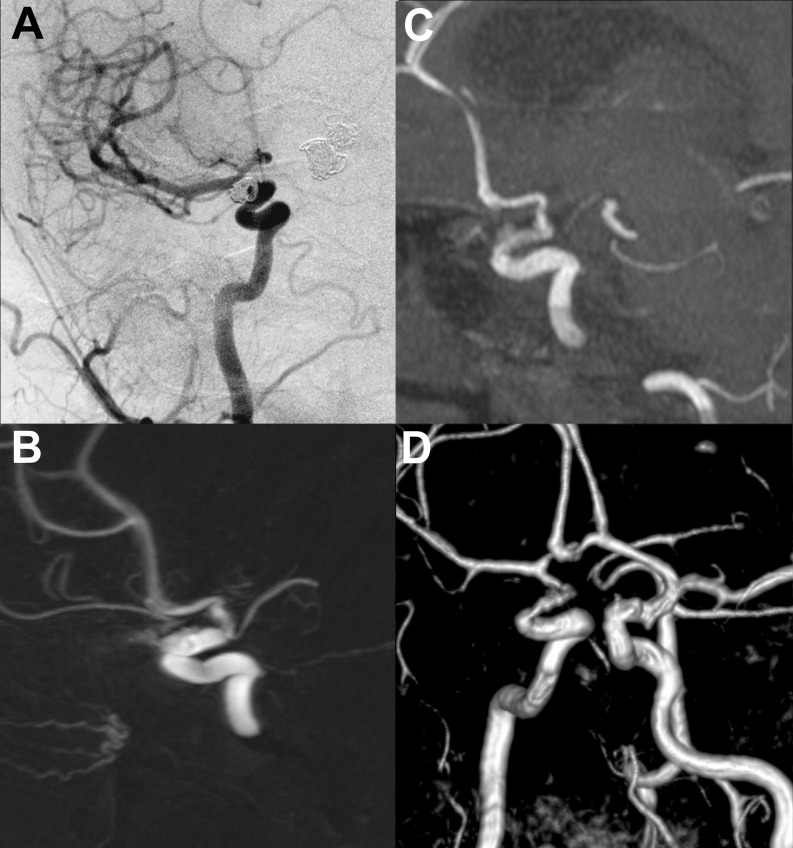
Follow-up angiography after stent-assisted coiling of the right internal carotid artery aneurysm. The 2D-DSAimage (**a**) and 3D-DSAMIP reconstruction (**b**) properly depict the non-stenosed stent lumen. There is a slight in-stent decrease in the signal intensity on TOF-MRA MIP image (**c**), which corresponds to false stenosis on TOF-MRA VR image (**d**)

TOF-MRA images were interpretable in all the cases. In two patients, DSA presented a protrusion of a coil loop into the parent artery, which was not visible on TOF-MRA images. There were no cases of branch occlusion and no significant susceptibility artifacts related to the coils. However, in all cases treated with stent-assisted coiling the signal intensity was decreased within the stent lumen, which imitated in-stent stenosis on volume-rendered images (Fig. 3). In such cases, the in-stent stenosis was not confirmed in either 2D-DSA or 3D-DSA(both presented normal flow within the stent and adequate depiction of the stent lumen). TOF-MRA correctly diagnosed complete occlusion in 42 patients and residual flow in 22 patients, while four diagnoses were false-positive and four were false-negative. The determination of the class of occlusion was incorrect in 11 patients. The decision on retreatment based on TOF-MRA was true-positive in 11 cases, false-positive in two cases, false-negative in one case, and true-negative in 58 patients.

The determination of the aneurysm occlusion status was the most accurate with 3D-DSA (*κ* = 0.92; CCC = 0.92, 95 % CI: 0.87–0.95), while 2D-DSA and TOF-MRA presented similar results (*κ* = 0.78; CCC = 0.85, 95 % CI: 0.77–0.90 and *κ* = 0.76; CCC = 0.80, 95 % CI: 0.70–0.87, respectively). Comparison of AUC values revealed only one statistically significant difference: 3D-DSA presented a higher diagnostic value than 2D-DSA in the determination of indications for retreatment (*P* < 0.050).

The most reproducible results of image analysis by one and two observers were noted in 2D-DSA followed by 3D-DSA and TOF-MRA (Table 4). Intraobserver agreement was very good for all techniques considering both the detection of the residual flow in the aneurysm and the possibility of retreatment (*κ* = 0.80–0.97). Classification of aneurysm occlusion status gave different results for 2D-DSA, 3D-DSA and TOF-MRA in 1.4 %, 2.8 %, and 6.9 % of cases, respectively. The respective percentages of differing interpretations of indications for retreatment were 1.4 %, 2.8 %, and 5.6 %. Interobserver agreement was moderate for TOF-MRA (*κ* = 0.72–0.74) and very good for 2D-DSA and 3D-DSA (*κ* = 0.80–0.94). Diverse results of aneurysm occlusion status evaluation with 2D-DSA, 3D-DSA and TOF-MRA were noted in 2.8 %, 5.6 %, and 12.5 % of cases, respectively. Respective proportions of different decisions on retreatment were 4.2 %, 6.9 %, and 8.3 %.

**Table 4:** Intraobserver agreement and interobserver agreement of index tests regarding the detection of the residual flow in the aneurysm and the decision on possible retreatment (*κ* with 95 % CI in parentheses)

	*κ*	2D-DSA	3D-DSA	TOF-MRA
Intraobserver agreement	Detection of the flow	0.97 (0.92–1.00)	0.94 (0.87–1.00)	0.86 (0.74–0.98)
	Decision on retreatment	0.95 (0.87–1.00)	0.92 (0.82–1.00)	0.80 (0.61–0.99)
Interobserver agreement	Detection of the flow	0.94 (0.87–1.00)	0.89 (0.78–1.00)	0.74 (0.59–0.90)
	Decision on retreatment	0.86 (0.71–1.00)	0.80 (0.64–0.97)	0.72 (0.51–0.93)

## Discussion

The risk of complications [21] and the high cost of DSA [22] raised the need for alternative imaging methods of follow-up after embolization of intracranial aneurysms. In previous studies, the diagnostic value of MRA has been determined versus various DSA techniques. Reference methods included 2D acquisitions in standard or working projections [10, 11], 3D-DSA [13, 14], or undefined combination of 2D and 3D-DSA [12, 23]. Despite numerous false results of 2D-DSA in the follow-up of embolized aneurysms presented by several authors [13–19], the method was not prospectively validated to our knowledge. In our study, the diagnostic value of 2D-DSA, 3D-DSA and TOF-MRA were tested against a retrospective consensus of 2D-DSA and 3D-DSA. Three-dimensional DSA presented the best diagnostic performance as concerns both the assessment of residual flow in the aneurysm and the decision on retreatment. The 2D-DSA and TOF-MRA had similar diagnostic values. An unexpected observation was the low value of 2D-DSA in the determination of indications for re-embolization.

The consecutive recruitment of patients scheduled for their first follow-up imaging at 3 months after the embolization resulted in collection of a relatively homogenous study population. This might reduce the influence of sample size on the results. The sizes and locations of the aneurysms were similar to those in other series of patients [3, 10]. The only significant difference was the low number of aneurysms in the posterior circulation in our material. This was caused by the system of bleeding aneurysm treatment in our center, where embolization is a method of choice.

In order to quantify the diagnostic value of intra-arterial arteriography we used the retrospective consensus of two observers simultaneously evaluating 2D and 3D-DSA images. In fact, we found no other reasonable method to noninvasively verify the performance of 2D-DSA, which is commonly considered the golden standard for follow-up. Combining multiple test results to construct a reference standard outcome including deterministic predefined rules, consensus procedures and statistical modeling is an accepted approach in cases when the reference test is imperfect [24, 25]. Generally, evaluation of a new modality which would be more sensitive than the contemporary standard usually causes methodological and statistical problems that result in a low specificity of the new method. Good examples of this dilemma are attempts to verify the value of 3D-DSA in follow-up. A recent systematic review revealed only three papers directly comparing 2D-DSA and 3D-DSA [26]. In the article by Kiyosue et al. [16], 3D-DSA presented as virtual endoscopy yielded the sensitivity and specificity of 100 % and 58.3 %, respectively. Buhk et al. [27], who tested 3D-DSA reconstructed as CT-like multiplanar reformations, reported a better diagnostic accuracy but considered this technique only as a supportive tool. The most comprehensive analysis was presented by Zhou et al. [17], who considered 3D-DSA as definitely more accurate and more reproducible than 2D-DSA despite the calculated moderate specificity and sensitivity of 3D-DSA vs. 2D-DSA used as a standard. In the three presented papers, the inappropriateness of classic test characteristics led to application of comparison statistics, including weighted kappa, chi-square test, and Pearson’s correlation coefficient, which are not able to valuate 3D-DSA [16, 17, 27].

According to our results, 3D-DSA should replace 2D-DSA as a reference method for follow-up, particularly in the process of decision-making on retreatment. The test characteristics of 2D-DSA for both the detection of the residual flow and the decision-making on possible retreatment were lower than those of 3D-DSA and comparable with those of TOF-MRA. The observed PPV of 2D-DSA indicates that only in 75 % of cases, the decision on retreatment was confirmed by the reference test. The low diagnostic value of 2D-DSA would have probably improved if working projections had been used. Working projections, which are used during embolization, are useful especially in detection of the aneurysm neck remnant [10]. However, a precise reconstruction of those projections at follow-up may be difficult even in center where embolization took place and may lead to an excessive exposition to radiation and contrast media. Still, when a precise 2D working projection is necessary, i.e., for re-embolization, a solution may be the use of 3D-DSA again. Several angiographic systems offer an easy c-arm positioning according to the 3D image presentation set by the user. This option is especially useful in retreatment of lesions located at the tip of the basilar artery or in the distal middle cerebral artery, where branch arteries may obscure the aneurysm neck.

In our series, TOF-MRA presented as an efficient follow-up method. Despite the lower values of test characteristics than that of 3D-DSA, the difference in the diagnostic value was not statistically significant when measured with AUC. Another important observation was only one false-negative result of TOF-MRA in the determination of indications for retreatment, which resulted in high NPV. This reflects very low probability of omitting clinically significant aneurysm remnant when using TOF-MRA. Our results confirm previous reports. In the meta-analysis by Kwee and Kwee [18], the pooled sensitivity and specificity of TOF-MRA in detecting residual flow was 83.3 % and 86.8 %, respectively. In a recent large prospective study by Schaafsma et al. [10], AUC of TOF-MRA was 0.86 (95 % CI, 0.81–0.91).

A significant drawback of TOF-MRA shown in our material was its inability to visualize coil loops protruding into the parent vessel, which was related to the use of 1.5-T field strength for MRA. Such a complication can be visualized with TOF-MRA at 3 T, as presented by Yoneoka et al. [28]. Another observed limitation was susceptibility to artifacts caused by implanted intracranial stents. Similar artifacts had already been observed by several authors [27, 29, 30]. In our patients, these artifacts resulted in false stent lumen narrowing on MR angiograms that might suggest in-stent thrombosis or proliferation of neointima [31]. Stent-related artifacts were most evident on volume-rendered images. In case of a true in-stent stenosis, one may expect a decrease of opacity or a filling defect on 2D-DSA images and the filling defect on 3D-DSA images. More advanced cases of stenosis may present as a difference in contrast medium flow proximally and distally to the stent in 2D-DSA, which reflects a hemodynamically significant blood flow disturbance. In neither of our patients, the stent lumen narrowing was confirmed by the reference test. Therefore, TOF angiograms should always be analyzed including source images and MIP reconstructions. None of the aneurysms treated with stent-assisted coiling presented residual flow. Thus, we may not discuss the possible influence of stent-related artifacts on determination of residual flow. This problem requires further investigation.

Variation in the assessment of aneurysms was reflected by the intraobserver reproducibility and interobserver agreement, which were significant. The 2D-DSA method presented the best agreement, which may be explained by its high resolution and limited possibilities of image adjustment. Since TOF-MRA was analyzed using unreconstructed images, as well as MIP and VR reconstructions, the variability of results was more evident. The observer agreements were at least comparable to previous reports [10, 12, 15, 27].

Study limitations included the mentioned above homogeneity of the sample, which was a result of the applied method of enrollment. We included consecutive patients who were coming for their first scheduled follow-up imaging visit and no active recruitment was carried out. Therefore, patients who died in the early postoperative period and those with severe neurological complications were lost from follow-up. Theoretically, those subjects might have presented a different rate of aneurysm remnants and altered cerebral hemodynamics that might have changed results of the study. Moreover, the method of enrollment limited the study population to patients with ruptured aneurysms only. However, we considered the study population appropriate because test characteristics of imaging modalities appear not to depend on the general patient condition and the preoperative aneurysm status [10]. As mentioned above, the reference test used in the study may be questionable as well. Nevertheless, in our opinion it was the only possible method to test the diagnostic value of 2D-DSA and 3D-DSA. Finally, we performed follow-up DSA examinations by administrating contrast medium into the CCA. At our institution, selective ICA angiograms are not routinely used in diagnostic procedures because we attempt to minimize the patient’s risk as much as possible. Selective catheterization of the ICA may increase the risk of vasospasm in young patients and may result in atherosclerotic plaque fragmentation in the older ones. On the other hand, cerebral vessel opacity might be increased with a selective ICA angiogram. However, in our experience, the used parameters of contrast media flow provide adequate depiction of arteries and of the residual aneurysm flow.

In conclusion, our results indicate that 3D-DSA is the contemporary golden standard for follow-up after aneurysm embolization. On the other hand, the relatively high diagnostic value of TOF-MRA favors this non-invasive method instead of intra-arterial DSA for the routine outpatient follow-up. The use of 3D-DSA should be limited to selected cases, which include patients with contraindications to MR, and those with uncertain MRA results after stent-assisted coiling or with coil protrusion. The 3D-DSA method should be also used to confirm indications for retreatment.
